# Development and Psychometric Validation of the Questionnaire for Social Communication in Young Autistic Learners (Q-SoCiAL)-Verbal Version

**DOI:** 10.1007/s10803-026-07444-8

**Published:** 2026-07-21

**Authors:** Katherine M. Walton, Alayna Borowy, Jolynn Pek, Dee Adedipe, Luc Lecavalier

**Affiliations:** 1 Nisonger Center, The Ohio State University, 1581 Dodd Drive, OHColumbus 43210, USA; 2 Department of Psychology, The Ohio State University, Columbus, OH, USA; 3 Department of Psychology, University of South Carolina, Columbia, SC, USA

**Keywords:** Social communication, Autism, Measurement, Questionnaire

## Abstract

**Purpose:**

There is a dearth of high-quality, efficient, and change-sensitive measurement tools for capturing social communication for young children with autism. The current study sets out to develop such a measure using best practices in patient-reported measure development.

**Methods:**

The Questionnaire for Social Communication in Young Autistic Learners (Q-SoCiAL) was developed in four phases: (1) construct definition and item generation using information from literature review and focus groups with parents, teachers, and experts; (2) item refinement via cognitive interviewing with parents and teachers of autistic children; (3) item selection and evaluation of structural validity using data from parents of autistic (*n* = 330) and neurotypical (*n* = 401) children; and (4) evaluation of external validity, including known groups comparisons (autistic vs. neurotypical children) and convergent/divergent validity evidence.

**Results:**

Preliminary analyses, cognitive interviewing, and expert review led to a final instrument containing 46 items appropriate for use with children who use more than 10 spontaneous words. Factor analysis on a sample of 330 children suggested a unidimensional structure. Internal consistency, test-retest reliability, and cross-rater consistency were very high. Q-SoCiAL scores correlated as expected with several clinical and demographic variables in the autism group.

**Conclusion:**

This new measurement tool fills an important gap in the field and has the potential to improve social communication measurement in clinical practice and research.

Challenges with social communication, including difficulty communicating wants and needs, challenges with forming friendships, and difficulty sustaining back-and-forth play interactions, are primary diagnostic characteristics of Autism Spectrum Disorder (hereafter autism; American Psychiatric Association [APA], 2022). While some of the differences in social communication observed in autistic children could be construed as *different* without being necessarily *problematic* (e.g., a preference for less eye contact; tendency to use more repetition in their language), a large proportion of young autistic children experience significant delays and challenges in social communication that clearly have a negative impact on their daily functioning. The type and severity of social communication challenges in autistic children range widely, and all autistic children (by definition) experience at least some clear social communication challenges. Autistic children who are most significantly impacted may struggle to communicate their basic wants and needs in an interpretable way, even to familiar caregivers. Children who are less impacted may be able to communicate in a variety of ways and for a variety of reasons, but struggle with adjusting social behaviors to different contexts or engaging successfully with same-aged peers (despite many autistic children having a high expressed desire for peer social interactions and friendships).

Given the prominence and impact of social communication challenges for autistic children, it is unsurprising that ensuring access to effective communication is prioritized and even categorized as a fundamental human right by both community members (e.g., autistic self-advocates, parents; Walton et al., 2024) as well as by researchers and clinical professionals who serve young autistic children (Camarata, 2022). Importantly, understanding and supporting the development of social communication is an especially important goal for young children who are still in the early stages of social communication development, for whom social communication difficulties are likely to have a profound impact on daily functioning and self-expression. Encouragingly, a variety of interventions for supporting social communication development in young autistic children have been developed and increasingly tested in high-quality clinical trials over the past 20 years (Sandbank, Bottema-Beutel, Crowley, Cassidy, Feldman et al., 2020b; Tiede & Walton, 2019).

Unfortunately, a dearth of high-quality, efficient, and change-sensitive measurement tools for capturing social communication is a major challenge that is limiting progress in this area (Anagnostou et al., 2015; Bishop et al., 2019). In their review of measurement tools for capturing change in social communication in autistic children, Anagnostou et al. (2015) did not identify a single tool that was deemed fully appropriate for use as a treatment endpoint. Tools identified as “appropriate with conditions,” all either had a limited developmental range, a high burden of training/administration, and/or limitations in measuring change over short periods of time. Parent- or teacher-report questionnaires (i.e., observer reported outcome measures [ObsROs]) tend to have the lowest burden of administration, making them easy to use in clinical practice and research. In addition, these measures allow observers to incorporate information from a range of settings and situations, which is important given that autistic children’s social communication may be highly context dependent. However, most social communication ObsROs have been used only in samples without developmental delays, are not appropriate for pre-school aged children, and/or contain few items that are appropriate for children with limited verbal language. Bishop et al. (2019) assert similar concerns in their commentary calling for a developmentally-based measure of social communication, including problems with using screening/diagnostic measures to track incremental differences in social communication; confounding of broad developmental level with social communication, particularly among early communicators; lack of sensitivity of existing measures to subtle changes over time; and over-focus on deficits rather than skill acquisition.

Thus, the current study set out to develop an ObsRO to measure early social communication in autistic children (ages 2–6 years old) that would address several of these needs and gaps, following best practice guidance for development and selection of Clinical Outcome Assessments (US Department of Health and Human Services FDA Center for Drug Evaluation and Research 2006; US Food and Drug Administration 2022b, 2022a). The first step of this process involved identifying which aspects of social communication were most important to patients and their representatives (via focus groups with parents, teachers, and clinicians). We also closely examined how clinical and educational experts defined early social communication as a concept of interest with regard to outcome measurement (via focus groups with clinicians and teachers, expert item review, and review of literature and existing measures). Second, response format, instructions, and an item pool were constructed based upon the inputs described above. Next, the item pool was refined via cognitive interviewing with parents and teachers to ensure questions and instructions were well-understood by participants. Finally, an item set was identified and structural (i.e., factor structure) and external (i.e., convergent and divergent, known groups) validity evidence were examined (Flake et al., 2017; Loevinger, 1957). The current manuscript will report on establishing and testing an item set most appropriate for children using at least some verbal language (i.e., greater than 10 words in current spontaneous vocabulary), the Questionnaire for Social Communication in Young Autistic Learners (Q-SoCiAL - Verbal Version); a future manuscript will identify and report on a version of the Q-SoCiAL that is designed for children who use 10 or fewer words (including preverbal children; i.e., Q-SoCiAL-Early Communication Version). Details of both the process and results from four phases of measure development (Phase 1: Construct Definition and Item Generation; Phase 2: Item Refinement; Phase 3: Item Selection and Evaluation of Structural Validity; Phase 4: Examination of External Validity) are described below.

## Method

### Phase 1: Construct Definition and Item Generation

Despite the importance of the construct of social communication for autism, there is (surprisingly) no single clear and accepted definition of the term within the research literature. The diagnostic criteria for autism categorize deficits in social communication and social interaction into three main types (social-emotional reciprocity, nonverbal communicative behaviors, and relationships) without clearly defining which skills fall under “social communication” versus “social interaction” labels (APA, 2022). Other theorists focused on early childhood have defined social communication skills much more specifically. For example, the Early Social Communication Scales (ESCS) categorizes skills on several dimensions including initiation versus response and function of communication (behavioral request, social interaction, or joint attention; Mundy et al., 2003). Theoretical models of adaptive behavior typically categorize “communication” and “socialization” as separate skill domains, with some measures (e.g., Adaptive Behavior Assessment System, 3rd Edition; Harrison & Oakland, 2015) placing communication skills under the broader domain of “conceptual” skills (along with academic skills and self-direction), while others (such as the Vineland Adaptive Behavior Scales, 3rd Edition; Sparrow et al., 2016) conceptualize communication as its own domain (comprised of expressive, receptive, and written). Notably, neither of these frameworks appear to fully capture the close interplay between social and communication skills (i.e., that communication is an inherently social act, and cannot happen outside of a social interaction) that is conceptualized in most other frameworks of social communication. Broader (not autism-specific) frameworks such as the Research Domain Criteria (RDoC) have also defined social communication as a key domain of functioning; the RDoC definition also acknowledges the close interplay of socialization and communication; the need to consider both productive and receptive aspects; and the use of both verbal and nonverbal forms of social communication (Insel, 2014).

In a recent large-scale meta-analysis of early autism intervention outcomes (Sandbank, Bottema-Beutel, Crowley, Cassidy, Dunham et al., 2020a), measures categorized as tapping social communication ranged from autism diagnostic measures (e.g., items from the Autism Diagnostic Interview-Revised and the Pervasive Developmental Disorders Behavior Inventory), items in various “social responsiveness” domains (e.g., social motivation, social awareness, or social communication on the Social Responsiveness Scale), scales purporting to measure “communication” (e.g., the Vineland Adaptive Behavior Scales communication domain), scales measuring “socialization” or “social skills” (e.g., the Vineland socialization domain; scales from the Social Skills Information System), as well as many more fine-grained measures tapping specific behaviors such as gaze aversion or number of gestures used on specific tasks. As Hansen et al. (2018) pointed out in their systematic review on measurement of early social communication, these disparate definitions and operationalizations pose serious concerns for understanding both the development of social communication in early childhood in a nuanced way, and for defining the effectiveness of social communication interventions, as intervention effectiveness appears to vary depending on how social communication variables are conceptualized. Thus, the first goal of the current measure development effort was to thoughtfully develop a definition of social communication to be used in the construction of this measure. We do not intend this definition to be “definitive” and do not expect that others will necessarily adopt this definition of social communication; instead, we hope that starting the measure development effort by clarifying the intended definition *to be used in the current measure* will enhance the substantive validity of the measure (Flake et al. 2017; Loevinger 1957; US Food and Drug Administration 2022b) by helping users determine whether there is a strong match between social communication as defined in the current tool and their own measurement purpose and identified concept of interest.

### Measurement Goals

Our team entered the measurement development process with several goals regarding formulation of a construct definition and resulting item pool. First, we aimed to create a measurement tool that would be useful for autistic children throughout the early developmental period (age 2–6 years). Thus, we aimed to understand and include key social communication skills and behaviors that were (a) likely to be displayed by autistic children between the ages of 2 and 6 years, (b) represented the range of language and communication abilities displayed by autistic children in this age range, including both verbal and preverbal children, and (c) reflect expert consensus regarding the most important aspects of early social communication. Second, we aimed to develop a tool that would have utility as a clinical outcome assessment with for tracking both short-term (e.g., 2–4 months) and long-term (i.e., 1 + years) change in social communication, given the types and durations of research and clinical interventions commonly used for this population. Thus, we wanted to ensure that our conceptualization of social communication (a) focused on skills likely to be targeted for and amenable to change, rather than more stable diagnostic features, (b) included skills viewed as important or impactful by end-users of the measure (i.e., parents, teachers, and clinicians), and (c) focused on skills for which improvement would likely be of some direct functional relevance or practical value to the child, rather than simply aiming for social normalization or aligning with autism diagnostic criteria. This final criterion is consistent with neurodiversity-informed conceptualizations of appropriate early intervention treatment goals, which emphasize that interventions should aim to build necessary communication, adaptive, and coping skills while de-emphasizing “passing as non-autistic” as a goal in and of itself (Ne’eman et al., 2023; Schuck et al., 2021). To accomplish these goals, we used multiple methods to construct a clear definition of social communication and identify a list of “facets” (i.e., specific sub-domains of social communication behaviors) that should be included to ensure appropriate item coverage across the full conceptualization of the construct.

### Focus Groups

A series of seven focus groups (*N* = 43) were used to identify social communication content important to parents, teachers, and experts. Results from these focus groups are reported in Walton, Borowy, & Taylor (2024) and thus will be described briefly here. Most participants conceptualized the social communication competencies and concerns for autistic children primarily through a skill acquisition lens and expressed a preference for strengths-based or competency-based measures. Regarding important skill areas reflecting social communication competencies, two major themes formed the bulk of the content discussed in the groups: social interaction (33% of all comments) and expressive communication (30% of all comments). There was also some discussion of receptive understanding (7% of content).

Expert clinicians were also directly probed about the differences between structural language skill and social communication; they consistently identified these as different (although related) constructs and offered examples of how non-autistic children (e.g., those with Down syndrome, specific language impairment, Deafness, or apraxia) frequently experienced substantial language delays in the face of relatively intact social communication. Despite strongly preferring a competency-based approach, parents (and to a lesser extent, teachers and expert clinicians) also mentioned several interfering behaviors that impacted their children’s social communication (e.g., emotion dysregulation, social overstimulation, limited communicative motivation; (Walton, Borowy, & Taylor, 2024).

### Review of Existing Measures and Measurement Frameworks

Prior to drafting items, we wanted to explore whether existing measures or item sets may adequately capture key aspects of the construct of social communication, and whether some items (or ideas captured by item sets) should be adopted for the current measure. Thus, a total of 70 questionnaire or interview measures tapping socialization, communication, or related constructs (e.g., adaptive behavior, challenging behavior, social emotional development) were reviewed. A total of 4,594 candidate items were compiled in a Microsoft Access database and then screened for relevance. Of the reviewed items, 4,205 were screened as potentially relevant to at least one of the social communication themes identified from the focus groups. The items were then sorted into broad themes as well as smaller “bins” of similar items (e.g., “using other people’s names” or “plays alongside peers”). This resulted in a total of 619 item bins falling under 141 sub-themes. The number of identified items per bin ranged from 1 to 35. While some content overlap with focus group themes was identified, most items focused on social deficits or symptoms rather than competencies. Competency-based items more often appeared on adaptive or language measures but were usually not consistent with the identified functional social communication emphasis of the current measure (e.g., items that focus on the number of words in a child’s vocabulary, rather than how these were used socially). Thus, rather than adopting items or items sets directly, the information from this collated and categorized item list was used to refine the construct definition and later to inform generation of specific items.

### Expert Review

Six experts with training in relevant disciplines including speech-language pathology and psychology and experience in pertinent areas such as clinical research, social communication, intervention, and scale development were consulted to assist with construct definition and item development. Experts gave written feedback on draft item sets and facets, and this feedback was discussed in more detail during a group meeting with all consultants. Consultants provided feedback on multiple elements of the draft measure including wording of response options, time frame, use of examples, and coverage of specific skill facets.

### Construct Definition

Information from focus groups, literature and measure review, and expert discussions were used to formulate the following definition of social communication for use in this measure (key terms in italics):

A child’s *frequency* and *independent use* of a variety of *verbal* and *nonverbal* behaviors *during interactions* with other people. These behaviors are used to *initiate*, *maintain*, *and/or respond* to others in ways that are *functional* (i.e., effective for achieving the child’s desired outcomes) and may serve a *variety of communicative purposes*, including requesting, sharing and exchanging information, fostering social connections, and achieving reciprocal social interactions.

Clarification: While some social communication skills will require verbal language and it is expected that scores on a measure of social communication would correlate with language ability, social communication as measured here is distinct from structural language skills (i.e., grammar, specific linguistic forms) and focuses more *on functional effectiveness of communication for social purposes.*

### Item Facets

Information from focus groups, measurement review, expert input, and review of several prominent definitions of early social communication (DSM-5-TR, ESCS, Adaptive Behavior as organized on the Vineland Adaptive Behavior Scales) were used to construct and refine a list of specific “facets” believed to be important to the construct of social communication as defined above. A full list of facets, a brief definition of each facet, and an example item from each facet appears in Table 1. Each facet’s level of representation within each data source (i.e., focus groups, existing measures, theoretical frameworks) is included in Table S1 in Supplemental Materials.

### Measure Structure and Response Options

Regarding the overall structure of the measure, focus group participants indicated that competency-based or strengths-based items were preferred to deficit-based. For example, an item reading, “My child uses words to clearly request an item” would be preferred to the item, “Has difficulties making simple requests.” This user preference for a competency-based measure led our team to structure the primary section of the measure with questions probing specific social-communication competencies, and how often children displayed these behaviors (a similar format to many adaptive behavior measures). In selecting response options, expert reviewers emphasized the need to make a distinction between ability (i.e., is not able; is able only with help) and frequency (uses the skill independently rarely, sometimes, or often). Focus group participants emphasized the importance of prompting and support for helping children perform new skills. Therefore, while most adaptive skill measures only credit behaviors performed independently, we elected to include an “only with help” option to capture supported skill use. Thus, we initially opted for a 5-point response scale that included the options, “**NEVER** does this behavior” “Does the behavior **ONLY WITH HELP**,” “Does the behavior without help **RARELY**,” “Does the behavior without help **SOMETIMES**,” and “does the behavior without help **OFTEN**.” Both experts and parents valued the variety of communicative modalities used by autistic children, particularly regarding potentially complex but non-vocal communicative forms, such as sign language and Augmentative and Alternative Communication (AAC) systems. Thus, instructions specifically prompt respondents to consider all forms of verbal communication, including vocal-verbal communication, sign language, and independent use of AAC systems, as equal.

### Item Generation

Once key facets for item generation and broad item structure were identified, the first two authors drafted potential items falling under each facet. The initial draft of items was reviewed and discussed by the panel of six expert reviewers for further revision. Focus group participants emphasized the need to clearly specify interaction partners for key social items, as children’s social behaviors typically differed substantially with different partners, particularly with regard to interactional differences with peers versus adults (Walton, Borowy, & Taylor, 2024). Therefore, we phrased most items to be specific to adult interaction (except items in the Peer Interaction facet). Across each facet, we aimed to include items with a range of difficulty and multiple communicative modalities to capture skills for autistic children across our target range of social communication skill (e.g., items about requesting included leading a parent by the hand; giving items; pointing; using words to request; and using phrases or sentences to request). This intentionally included items that tapped into unconventional forms of communication (e.g., using another person’s finger to point to an item; flapping hands to show happiness) as positive signs of social communication competency. This resulted in an initial item pool that contained 103 items, which was used during cognitive interviewing.

### Phase 2: Item Refinement

#### Cognitive Interviewing

Thirty parents and 29 teachers of autistic children in the targeted age range (Child Age in months: *M* = 57.1, *SD* = 14.3, Range = 30–82) participated in cognitive interviews using a respondent debriefing strategy, with responses organized using the problem coding frame proposed by Presser and Blair (1994). All interviews were conducted virtually. Both parents (*n* = 30) and teachers (*n* = 29) were predominately female (96% parents; 79% teachers), identified as white (73% parents, 72% teachers) and non-Hispanic (83% parents, 97% teachers), and were reporting about autistic boys (67% parents; 90% teachers). Each participant completed a subset of 38–40 questions from the item pool immediately before the interview, such that each individual item received feedback from a minimum of three parents and three teachers. Participants who reported that their child had limited language skills (i.e., preverbal or single words) were given only items relevant for children with limited language to increase applicability of the item set for individual children. The interviewer then reviewed the questions one by one with each participant and asked them to talk through their decision process for selecting their response, and to identify any parts of the question that were confusing. Participants were also asked to provide feedback on the overall structure and content of the item pool (e.g., any key topics that were missing) and on the instructions. Changes to the item pool resulting from cognitive interviews involved several modifications, including shortening and simplifying the initial instructions based on participant feedback that they were too long. In addition, an “outgrown” response option was added, as several participants reported confusion about how they should answer regarding early-emerging developmental skills (e.g., requesting using a pointing gesture) for a child who is now using a more complex skill to serve the same communicative function (e.g., requesting using full sentences). This confusion occurred frequently despite measure instructions including clarification that respondents should rate skills that have been outgrown as “often.” A total of 46 new or restructured items were added based on participant feedback, particularly on the Conversation facet. In addition, 80 items underwent minor changes, such as wording changes or addition of examples. The utility of existing items was reinforced, with strong parent and teacher enthusiasm about the strengths-based, fine-grained approach to understanding their child or student’s social communication.

#### Final Item Pool for Use in Structural Validation Studies

The final item pool comprised 149 items across 21 facets. The child’s social communication skills and behaviors are assessed over the past month. Items are rated by the respondent on a 6-point Likert-type scale, corresponding to the child’s independence (i.e., with or without help) and frequency of behavior, which is ultimately collapsed to a 4-point (0–3) scoring scale. Responses of “never does this behavior,” and “does the behavior only with help” receive a score of 0 points (the decision to collapse these scale points was based on infrequent use of the “only with help” response option); responses of “does the behavior without help rarely” receives 1 point; “does the behavior without help sometimes” receives 2 points; and “does the behavior without help often” and “has outgrown the behavior” each receive 3 points. For any item requiring verbal skills, a reminder prompt is included below the item that reads, “Includes words, signs, or use of a communication device” to ensure that respondents give children appropriate credit for non-spoken forms of spontaneous verbal communication.

### Phase 3: Item Selection and Evaluation of Structural Validity

#### Participants and Procedures

Parents of autistic children were recruited online through the Simons Foundation Powering Autism Research Knowledge (SPARK) Research Match System. The first round of data collection took place between June and September 2023 (including all test-retest and cross-rater data), and additional data from 154 girls at a single time-point were collected in May 2025 to increase sample size for autistic girls. Parents of potentially eligible autistic children (based on current child age between 2 years, 0 months and 6 years, 11 months) were sent an email invitation (and up to three reminders if necessary) to participate in the study. A total of 3,373 invitations were sent. Of these, 526 (15.6%) consented to the study and 510 (96.9% of those consented) provided at least some data. Unfortunately, the total number of preverbal children (i.e., those that indicated their child did not regularly use spoken words to communicate and/or had 10 or fewer total words in their spontaneous vocabulary) in the sample was relatively small, which limited our ability to fully understand the utility of scores on items most useful for preverbal children. Thus, the analyses reported in this paper will focus on psychometric properties of the Q-SoCiAL version intended for verbal children (i.e., any child reported to use at least single words regularly and regular use of greater than 10 different spontaneous words). A future study will focus on further examination of items most useful for preverbal children.

From the original 510 participants who began the survey, 180 were excluded from all remaining data analysis due to missing data (*n* = 82), being preverbal (*n* = 30), or using 10 or fewer words (*n* = 68). During the first round of data collection, parents were asked whether they would be interested in completing the survey again approximately two weeks later, whether they were interested in referring a second parent/primary caregiver for participation, and whether they were interested in referring an educator (e.g., teacher, therapist, childcare provider) for participation. Seventy-three children who used greater than 10 words were rated twice for test-retest, 39 for parent-parent concordance, and 37 for parent-teacher concordance.

All parents completed a brief demographic form to collect information both about themselves and the child about whom they were responding. Parents and educators who provided information as a second rater completed a demographic form only about themselves (child demographic information from Rater 1 was used for child data). All respondents then completed the full item bank from the draft Q-SoCiAL measure as described above (149 items). Demographic information for children and raters in each group is reported in Table 2. While the full sample (*n* = 330) collected online had an equal number of boys and girls, children in all three subsamples (test-retest, parent-parent, parent-educator) were more likely to be male compared to the full autism sample, as paired and retest samples were not recruited during the second wave (which only recruited autistic female participants).

## Results

### Selection of Preliminary Item Set

First, intra-class correlations using a two-way mixed-effects, absolute agreement model were calculated to quantify item-level test-retest correlations for each individual item (Koo & Li, 2016). Thirty-three of the 149 items had item-level test-retest ICCs below 0.60, and another 57 items had ICCs between 0.60 and 0.70. Items with ICCs below 0.70 were examined for further indicators of low item quality, including low item-total correlations, low variability, floor or ceiling effects, or unusual response patterns across language levels suggesting rater misinterpretation of items. We removed 41 items that had low ICCs and also had at least one other concern related to item variability or distribution (item-total correlation < 0.50, mean above 2.5 or below 0.5 on a 0–3 scale, standard deviation below 0.75, odd response patterns across language levels). An additional 11 items with low ICCs (below 0.60) were also removed even though there were no additional clear indicators of low quality. This left 97 competencies items in the analysis pool.

Bivariate Pearson’s correlations (recommended as appropriate for ordinal data by Norman, 2010) were run among the remaining 97 items to detect highly-correlated item pairs that may be redundant and could be considered for elimination. A total of 16 correlations at or above 0.80 were detected, primarily between items within a facet. High correlations especially clustered in a few facets, including “Seeking Information” (5 correlations > 0.80 among 7 items) and “Telling about Past Events” (4 correlations > 0.80 among 5 items). The remaining items were examined with attention to high correlations, redundant content, item difficulty, item-level ICC, and balance of number of items in each facet. The goal was to retain at least 1–2 items from as many facets as possible, while ensuring selection of items across the difficulty range with favorable individual item properties that would also be useful for children with language ranging from single words to complex speech. This item trimming led to a total of 46 items being retained for further analysis.

### Exploratory Factor Analysis

An exploratory factor analysis (EFA) was conducted to examine the factor structure of the instrument using the *lavaan* package in R version 4.5.1 (Rosseel, 2012). Given the ordinal nature of the item responses and the violation of the multivariate normality assumption, the Weighted Least Squares Mean and Variance adjusted (WLSMV) estimator was employed. The WLSMV estimation was designed for ordinal data, using polychoric inter-item correlations accompanied by robust standard errors and test statistics for EFA (Muthén et al., 1997). An oblique Quartimax rotation was utilized to allow for potential correlations among the factors (Browne, 2001). Clinical interpretability and model goodness-of-fit indices were used to determine the optimal factor solution. These indices included the Comparative Fit Index (CFI; Bentler, 1990), Standardized Root Mean Square Residual (SRMR; Hu & Bentler, 1999), Root Mean Square Error of Approximation (RMSEA; Browne & Cudeck, 1992), and Tucker-Lewis Index (TLI; Tucker & Lewis, 1973). Recommended cutoffs for acceptable model fit, as outlined by Hu and Bentler (1999) and Schreiber et al. (2006) are: CFI and TLI ≥ 0.90, SRMR ≤ 0.10, and RMSEA ≤ 0.08.

Prior to conducting the EFA, the number of factors to extract was assessed using multiple criteria, including the scree plot, eigenvalues, and parallel analysis. The first seven eigenvalues of the correlation matrix were 26.64, 3.23, 1.66, 1.22, 1.12, 1.05, 0.95, with the first eigenvalue approximately eight times larger than the next eigenvalue and explaining approximately 60% of the total variance. Parallel analysis suggested retaining up to three factors, and the scree plot indicated a marked leveling off of the curve after the third factor. Thus, one- to three-factor solutions were subsequently examined.

The one-factor solution demonstrated the best clinical meaningfulness and acceptable model fit, with RMSEA = 0.079 (90% CI: 0.076 – 0.082), CFI = 0.948, TLI = 0.945, and SRMR = 0.083. The two- and three-factor solutions lacked clinical interpretability and meaningful explanatory value beyond item difficulty (i.e., more difficult versus easier items clustered together on 2-factor solution), and several items exhibited cross-loadings across multiple factors, some of which demonstrated nearly equal loadings on multiple factors. Therefore, the unidimensional solution was retained as the optimal factor structure. Abbreviated item stems and factor loadings are included in Table 3. All factor loadings were significant at *p* < .0001. Fit statistics for the 1-, 2-, and 3-factor solutions are reported in Table S2 in supplemental materials.

### Sum Score Distributions

**Fig. 1:**
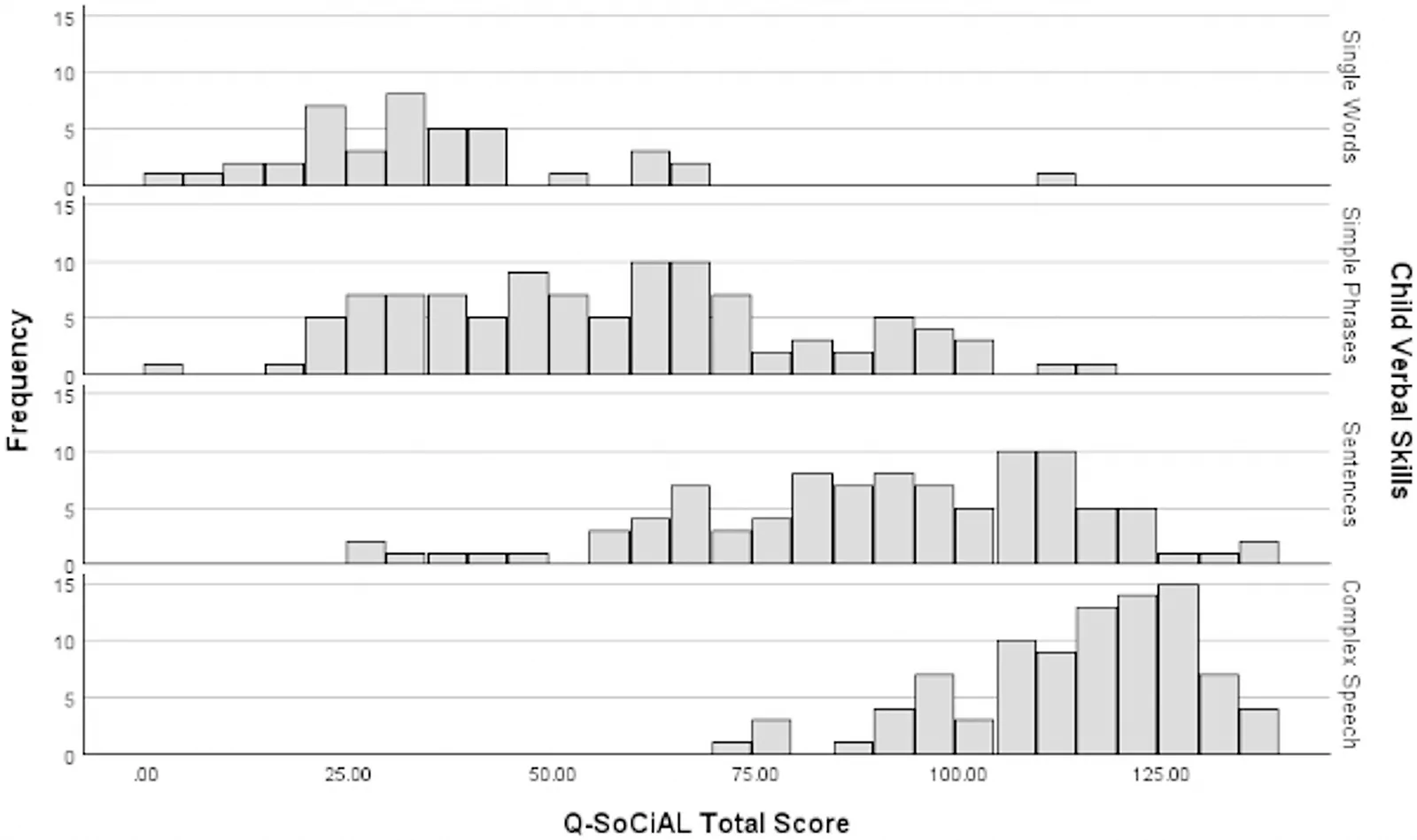
Histogram of Q-SoCiAL score by language level. *n* = 41 single words; *n* = 102 short phrases; *n* = 96 sentences; *n* = 91 complex speech

Descriptive statistics were calculated for sum scores on the 46-item measure using data from all *n* = 330 children with complete data from the initial respondent. Scores could theoretically range from 0 to 138. Children’s sum scores ranged from 2 to 138 (*M* = 80.0, *SD* = 35.0) and were clearly not normally distributed upon visual inspection. When splitting children by language level (single words, simple phrases, sentences, complex speech as reported by parents on a single item), scores within each language level more closely approached normal distributions (particularly among children with simple phrase speech), but non-normality was still clear among both the lowest language group (with notable positive skew) and the two higher language groups (with notable negative skew, especially among children with complex speech). Scores differed as expected by child language level, with children who had more complex speech attaining higher scores, although still with considerable variability within groups and substantial score overlap between groups (Single Words *M* = 34.9, *SD* = 19.6, Range 2–111; Phrase Speech *M* = 57.2, *SD* = 23.9, Range 4–118; Sentences *M* = 90.8, *SD* = 24.3, Range 26–137; Complex Speech *M* = 114.4, *SD* = 14.6, Range 73–138). See Fig. 1 for histograms separated by language level. On a 0–3 scale, individual item difficulties ranged from a mean score of 0.79 (*SD* = 1.03) for the most difficult item (“Actively maintains a conversation for at least 2 minutes by commenting, answering, or asking questions”) to a mean of 2.69 (*SD* = 0.69) for the easiest item (“Names toys or pictures using single words”). Item-level difficulty information is reported in Supplemental Table S3.

### Correlations With Demographic and Clinical Variables

Correlations with demographic and clinical variables were calculated using the full online sample (*n* = 330). Given the focus on the measure on skill acquisition, sum scores were strongly positively correlated with the child’s verbal level as rated by the parent, *r* (328) = 0.792, *p* < .001, and more modestly but significantly positively correlated with child chronological age, *r* (328) = 0.262, *p* < .001. Scores were uncorrelated with child sex, *r* (328) = 0.089, *p* = .11, parent age, *r* (319) = 0.075 *p* = .18, or family income, *r* (315) = 0.073, *p* = .20, but were weakly positively correlated with parent education, *r* (326) = 0.188, *p* < .001. However, the correlation between parent education and sum scores disappeared after controlling for child language skills, *r* (322) = 0.003, *p* = .959.

### Internal Consistency

Internal consistency for the 46-item measure was excellent (α = 0.98). Item-total correlations ranged from 0.26 to 0.85, with lower item-total correlations occurring among the easier items on the scale that had high endorsement of the top (Often or Outgrown) scale point. See Supplemental Table S3 for all item-total correlations. Pearson’s correlations among the 46 items ranged from 0.06 to 0.85, with a mean inter-item correlation of 0.45.

### Test-Retest Reliability

Test-retest reliability was calculated using scores produced by the subset of 73 parents who completed the measure at two time points approximately 2 weeks apart (an interval across which little meaningful change would be expected to occur), and had no missing items used in the sum score at either time point. Test-retest reliability (average of 12 days) produced an ICC = 0.96, suggesting a high level of stability in sum scores. Test-retest ICC for individual items ranged from 0.66 to 0.89, with a mean item-level test-retest ICC of 0.75.

### Cross-Rater Consistency

Parents who completed the measure online in Wave 1 of data collection were encouraged to invite a second caregiver and/or the child’s educator (teacher, therapist, childcare provider) to also complete the measure to examine cross-rater consistency. Complete data for verbal children was available from 39 parent-parent dyads and 37 parent-educator dyads. Educator raters were nominated by the parents and included childcare center teachers (19%), nannies/babysitters (27%), general education teachers (11%), special education teachers (24%), Applied Behavior Analysis providers/therapists (14%) and family/friends who provided regular care for the child (5%). Cross-rater consistency between 2 parents (ICC = 0.83) and between parent and educator (ICC = 0.86) was very high, suggesting that different raters, including raters who observed children in different contexts, were able to produce scores with a high level of agreement.

### Phase 4: Examination of External Validity

#### Known Groups Validity

##### Participants and Procedures

Parents or primary caregivers of children without known developmental differences were recruited through a variety of panels engaged by Qualtrics recruitment services. Caregivers were asked, “Has your child been diagnosed with a disability or other condition that significantly impacts their communication or social interaction (e.g., autism, Down syndrome, speech/language delay)?” Caregivers who answered “yes” to this question were screened out. In addition, caregivers were asked as part of the child demographic form, “Does your child have any medical, developmental, or behavioral diagnoses that affect their daily functioning?” If caregivers endorsed a significant developmental condition potentially impacting communication (e.g., speech/language disorder, autism, cerebral palsy) their data were not included. Parents indicating medical conditions unlikely to impact social communication (e.g., asthma) were included in the sample. Data were also screened for low quality (e.g., speeded responses, straight-line responding, duplicates). A total of 401 caregivers of children with some verbal language and more than 10 words in their spontaneous vocabulary completed the questionnaires. Participating caregivers (91% biological parents, 2% adoptive parents, 7% others, including foster parents, grandparents, and stepparents) completed a demographic survey and the 149-item draft Q-SoCiAL to report on their children’s skills. The majority of responding caregivers identified as female (82%), white (80%) and non-Hispanic (90%). Just over half (53%) of the children being reported about were girls, and they ranged in age from 14 to 71 months (*M* = 46.3, *SD* = 14.7). Roughly equal numbers of children fell in the three, four, and five-year-old age bands (*n* = 97 three-year-olds; *n* = 99 four-year-olds; *n* = 96 five-year-olds). A slightly smaller proportion comprised two-year-olds (*n* = 82; 20%), and the smallest proportion of children fell in the one-year-old age band (*n* = 27; 7%); these smaller numbers of younger children were due to more children in the younger age bands being excluded from the current analyses because they currently use 10 or fewer words (initial recruitment yielded ~ 100 children per age band). See Table 2 for additional demographics.

#### Analyses and Results

**Fig. 2:**
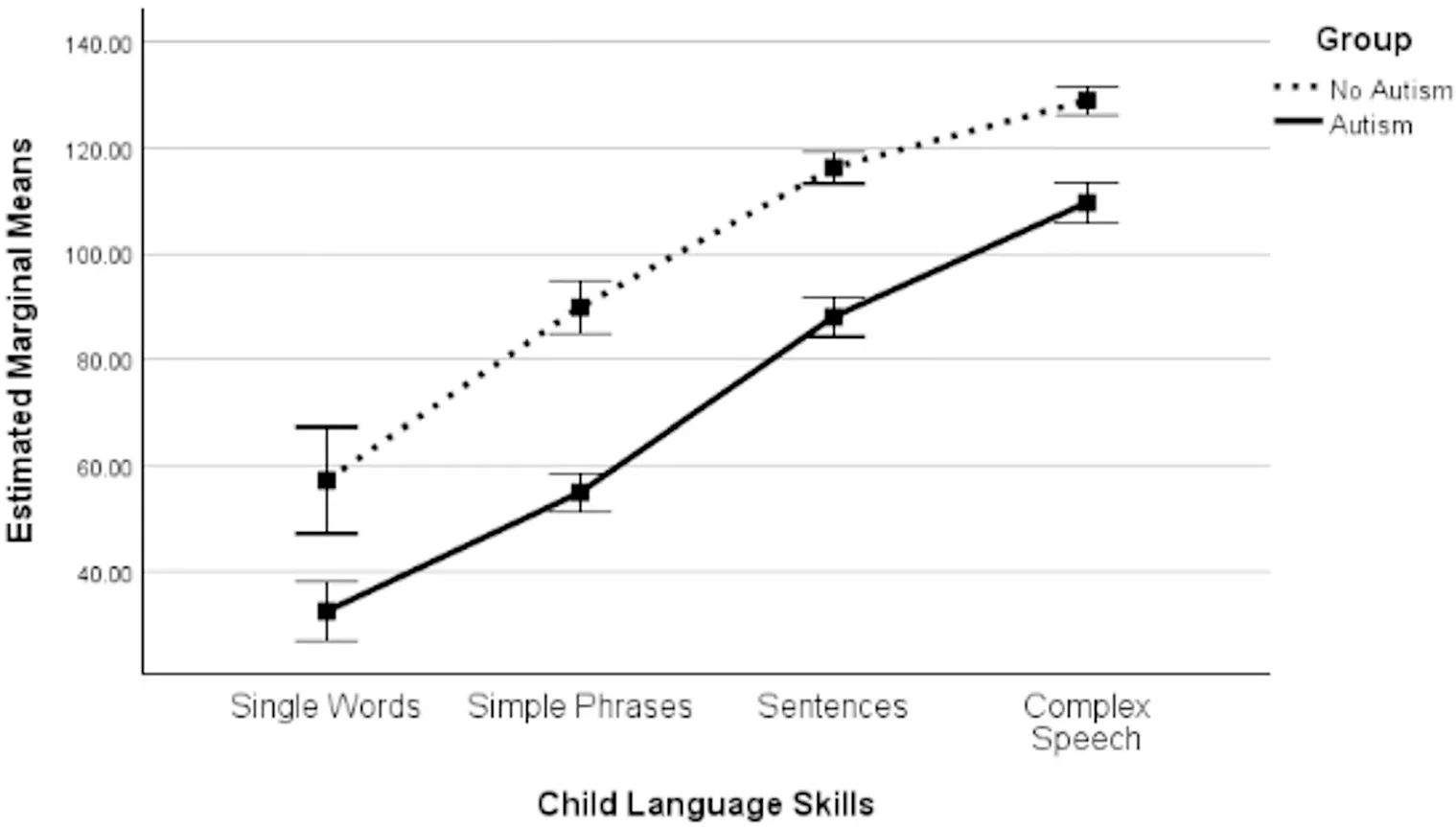
Q-SoCiAL score by language level and group. Error bars = 95% Confidence Interval. Covariates appearing the model are evaluated at child age = 53.07 months

Two-way ANCOVA was used to examine differences between sum scores on the 46-item Q-SoCiAL -Verbal Version in a group of autistic children and children without known developmental differences, controlling for chronological age. Chronological age in months was entered as a covariate, while group (autism vs. non-autism) and language level (four-point scale; single words, simple phrases, sentences, complex speech) were entered as fixed factors. The ANCOVA indicated main effects of age, *F* (1, 722) = 47.74, *p* < .001, language level, *F* (3, 722) = 272.93, *p* < .001, and group, *F* (1, 722) = 151.41, *p* < .001. There was also a significant group x language level interaction, *F* (3, 722) = 6.01, *p* < .001. All pairwise follow-up tests were significant at the *p* < .001 level after employing Bonferroni corrections. Examination of the group x language level interaction suggested that group differences were smaller (but still present) in the highest-language group, likely due to truncation of scores for non-autistic children with the highest language skills, whose group mean was near ceiling. See Fig. 2 for age-adjusted mean scores by group and language level.

### Evidence for Convergent and Divergent Validity

#### Participants

Nineteen autistic children who currently used greater than 10 words completed an in-person clinic visit with a parent (2 fathers, 17 mothers) to complete several measures of development, social and communication skills, and autism-related characteristics to examine convergence and divergence of the Q-SoCiAL-Verbal Version with other well-validated measures. Children ranged in age from 43 to 82 months (*M* = 61.23, *SD* = 13.89) and most children were male (84.2%). Parent-reported verbal skills included children with single words (*n* = 3, 15.8%), simple phrases (*n* = 6, 31.6%), sentences (*n* = 6, 31.6%), and complex speech (*n* = 4, 21.1%). See Table 2 for additional demographics.

#### Procedures and Measures

Children completed assessments in-person in a clinic room; testing was completed either individually with a clinician or with a parent present depending on parent and child comfort. When convenient for families, parent questionnaires and interview measures were completed virtually over a videoconferencing platform. Parents completed a demographic form, the draft 149-item Q-SoCiAL measure, the Behavior Inflexibility Scale (BIS; Lecavalier et al., 2020), and the Vineland Adaptive Behavior Scales, 3rd Edition Parent Interview form (VABS; Sparrow, Cicchetti, & Saulnier, 2016). Children completed a measure of cognition (Stanford Binet Intelligence Scales, 5th Edition Early Childhood Version; SB-5; Roid, 2003) and participated in a ~ 15-minute semi-structured play interaction with the clinician that presented a variety of social and communication presses as well as free play opportunities. Parents and children were also videotaped playing together for 10 min. An expert clinician (first author) rated the Childhood Autism Rating Scales, 2nd Edition (CARS-2; Schopler et al., 2010) based on all collected clinical data *except* the Q-SoCiAL.

#### Analyses and Results

Correlational analyses between the Q-SoCiAL-Verbal score (sum of 46 retained items) and measures of convergence (VABS Socialization, VABS Communication, CARS-2) and divergence (VABS Daily Living, VABS Motor, BIS) were conducted both as zero-order correlations, and as partial correlations controlling for SB-5 Nonverbal IQ. Given the structure and developmental ordering of the Q-SoCiAL items, we expected zero-order correlations between Q-SoCiAL scores and other measures tapping general development (i.e., SB-5 scores and all VABS domains) to be relatively strong as they share meaningful variance with regard to overall developmental level and all are expected to increase with age and development. Pearson’s correlations between Q-SoCiAL scores and SB-5 IQ scores were significant and moderate (*r* (17) = 0.59, *p* < .01 for Nonverbal IQ; *r* (17) = 0.64, *p* < .01 for Verbal IQ; *r* (17) = 0.65, *p* < .01 for Full-Scale IQ). Q-SoCiAL was also moderately and significantly correlated with VABS Communication and Socialization domain scores, and strongly negatively correlated with CARS-2 t-scores. In contrast, Q-SoCiAL scores showed small and non-significant correlations with VABS Daily Living and Motor domain scores and with the BIS. When controlling for SB-5 Nonverbal IQ, Q-SoCiAL scores remained significantly correlated with VABS Socialization and CARS-2 scores but were no longer significantly correlated with VABS Communication. See Table 4 for correlations between Q-SoCiAL and other variables of interest.

## Discussion

This report describes the development and validation of the Q-SoCiAL-Verbal Version, a questionnaire measure of social communication for autistic children ages 2–6 who use more than 10 spontaneous words. This 46-item, parent-report measure focuses on understanding children’s acquisition and independent use of functional social communication skills likely to be targeted during early delivered interventions for autistic children (e.g., behavioral and NDBI therapies, speech therapy, social skills groups). It is intended to be used by a variety of clinicians and/or clinical researchers who are interested in obtaining a fine-grained picture of early social communication in autistic children, understanding within-sample variability in social communication, or potentially measuring change following social communication intervention (although sensitivity to change has not yet been tested in longitudinal or treatment studies). This new measurement tool fills an important gap in the field, as existing measures of early social communication suffer from limited construct coverage, high burden of administration and scoring, focus on diagnostic features rather than treatment-relevant skills, and limited item coverage for children who are early in their use of verbal language (Anagnostou et al., 2015). Initial psychometric evaluation of the Q-SoCiAL-Verbal Version indicates (a) excellent test-retest reliability, cross-rater consistency, and internal consistency, (b) a relatively unidimensional structure that lends itself to simple sum scoring for clinical convenience, (c) ability to capture variability in skills that goes beyond simple structural language, and (d) promising initial indications of concurrent validity with well-established measures of social and communication functioning.

To make meaningful advancements in clinical care for young autistic children, it is essential to move toward conducting studies that focus on real-world effectiveness of therapeutic supports in community settings. Conducting clinical trials and other clinical outcome studies in such settings requires use of outcome measures that capture important and treatment-relevant aspects of the social communication construct, are quick and efficient to administer by non-experts, can capture change over time, and are psychometrically sound (Bishop et al., 2019). The Q-SoCiAL was developed specifically with these needs in mind. First, care was taken to thoughtfully define and operationalize the construct of early social communication as it is relevant to young autistic children to maximize applicability for early intervention researchers and clinicians. The measure is competency-oriented, with a focus on social communication behaviors that have practical and functional relevance for autistic children in their daily lives. Importantly, items on the measure were designed to capture multiple forms of expression, including autism-specific forms of expression, and intentionally avoided including items that were overly focused on performing neurotypical forms of social expression (e.g., making “appropriate” eye contact) or “passing” as non-autistic. The tool is intended to cover a wide range of early social communication behaviors, covering behavior across 21 expert- and parent-identified facets of social communication that are common targets for early intervention, including social play, joint attention, requesting, imitation, conversation, and social cognition (although each is covered with only a few items, and thus the Q-SoCiAL would likely need to be paired with other curricular measures to be useful for treatment planning). While a single measure cannot cover all potentially relevant aspects of social communication, content selections were informed carefully by experts, parents, and teachers. By providing a clear description of how content was selected and what content is included, potential users can determine whether this operationalization of social communication is a suitable fit for their intended purposes. Importantly, the Q-SoCiAL is practical for use in community settings, as it can be completed independently by an untrained parent, teacher, or clinician in less than 10 min.

The Q-SoCiAL also has several key properties that suggest it may be promising as a measure of change, although further testing in longitudinal or treatment studies will be needed to confirm its sensitivity to change. First, it was iteratively developed using a range of input from end-users and experts, with sensitivity to change and treatment-relevance of the items guiding the development of the measure from the start. Second, Q-SoCiAL scores demonstrate excellent reliability, a key prerequisite to change sensitivity. In particular, scores showed a very high level of stability across a two-week test-retest period, a period during which little meaningful social communication change is expected to occur. Moreover, scores showed high cross-rater consistency, both between two parents and between a parent and an educator. This suggests that scores on the items capture behaviors that are stable and observable across settings. Alongside this high degree of test-retest and cross-rater consistency, scores also showed substantial variability within a sample of autistic children, with children in the initial sample of 330 scoring across the entire possible range of the measure. While scores on the Q-SoCiAL correlated strongly with rough parent ratings of overall child verbal ability, scores within each verbal scale point still varied widely, with a range of more than 100 points within most individual language-level groups (and a minimum range of 65 points within any given scale point). This suggests that the limited range of available scores on the parent-rated verbal item concealed substantial within-language-level variability that is likely to be clinically meaningful. While 46 items is a relatively large number for a single-factor measure, the inclusion of items across a relatively wide ability range may facilitate the usefulness of the tool across the heterogeneous population of children likely to be enrolled in early autism intervention studies. This item set also intentionally includes theoretically meaningful item coverage across the range of identified social communication facets. Finally, in a small sample, the Q-SoCiAL-Verbal also demonstrated positive correlations with other more time-intensive measures of related constructs, including some that have been used to measure change in previous treatment studies (e.g., the Vineland Adaptive Behavior Scales). An additional notable strength is that item selection activities were performed using a sample that included an even balance of autistic boys and girls, which is important given that some studies have found gender differences in specific aspects of early autistic social communication (e.g., play, facial expressions; Beggiato et al., 2017).

### Limitations and Future Directions

These promising initial findings should be extended in several ways. First, several additional steps are needed to confirm and extend the utility of the Q-SoCiAL-Verbal Version for use in treatment trials. While the measure was designed with sensitivity to change in mind, the Q-SoCiAL’s ability to capture change over time has not been tested directly. It will be essential for future studies to examine responsivity to change over time and following intervention to confirm its utility as an intervention outcome measure. In addition, only preliminary data with a very small sample of children has been gathered to examine evidence for convergent and divergent validity. Future work should build on this to understand the extent to which the Q-SoCiAL measures overlapping or distinct constructs associated with other commonly used measures of early development, socialization, and language. Future work should also more closely examine how the measure performs across different groups of children (e.g., girls versus boys; non-native English speakers; different racial and ethnic groups) to examine whether the Q-SoCiAL retains measurement invariance (i.e., same factor structure) and similar reliability indicators (i.e., test-retest, cross-rater consistency) across different sub-populations of autistic children.

Second, the data presented in this manuscript focus on parent-reported skills for children with more than 10 functional words in their vocabulary (which, in our sample, comprised ~ 77% of the autistic children recruited). Understanding social communication variability and measuring social communication change in children who are not yet using functional language is an extremely pressing need, and a version of the Q-SoCiAL appropriate for preverbal children is also important; additional data collection to inform selection and validation of an item set for a Q-SoCiAL-Early Communication version is currently in progress. In addition, an educator-report version of the Q-SoCiAL (for use by teachers, childcare providers, or clinicians as respondents) was developed alongside the parent-report version. The parent-reported and educator-reported version showed substantial convergence (at least for the 46-item Q-SoCiAL-Verbal version) in a small sample, suggesting that this measure can also be completed by teachers or clinicians; however, it is important to further validate the educator report version of the Q-SoCiAL to understand how the tool might be used in educational and clinical settings. Finally, additional item analyses (e.g., using item response theory) are likely to be useful to identify the most appropriate items for short-forms of the Q-SoCiAL, as well as to identify scoring algorithms that can be used to equate scores obtained from Q-SoCiAL – Verbal version and the forth-coming Early Communication version.

Finally, more sophisticated scoring algorithms that benchmark scores to normative data and assist with identifying a minimally clinically important difference or meaningful score regions will be helpful in extending the use of the Q-SoCiAL for additional settings and applications, including potential use as a clinical outcome measure. It is possible that the Q-SoCiAL may also be useful for children with non-autism developmental challenges (e.g., Down syndrome, developmental delays) and understanding the extent to which Q-SoCiAL scores track autism-specific social communication challenges versus more general developmental delays in social communication will be informative.

## Conclusion

The Q-SoCiAL-Verbal is a novel, parent-reported questionnaire measure of early social communication intended for autistic children ages 2–6 years old who use more than 10 spontaneous words. It has been carefully developed using input from end-users and experts to capture treatment-relevant social communication skills and shows excellent reliability and positive early indicators of construct validity. The Q-SoCiAL fills an important gap in efficient, fine-grained measurement of early social communication skills in young autistic children. Researchers or clinicians interested in using the Q-SoCiAL should contact the author.

## Supplementary Material

Appendix A_Measure List

Table S3_Item Characteristics

Table S2_Fit Statistics

Table S1_Item Facet Coverage

**Supplementary Information** The online version contains supplementary material available at https://doi.org/10.1007/s10803-026-07444-8.

## Figures and Tables

**Table 1:** Facet list, definitions, and example items

Facet and definition	Example item

*Requesting*	
Intentional efforts to communicate wants or needs with others.	Shows someone what they want by giving an item (Example: handing a parent a cup to ask for water).
*Commenting*	
Describing and labeling objects, actions, or events.	Describes objects or pictures using sentences with four or more words (Example: “The red ball is bouncing.”)
*Sharing Interest*	
Intentional efforts to direct another person’s attention to an object, event, or topic of interest (includes conventional indications of joint attention).	Points at something to share their interest with an adult (Examples: a dog walking by or a picture in a book).
*Gaining Attention*	
Intentional efforts to elicit another person’s attention using verbal or nonverbal means.	Says “mama,” “dada,” or another caregiver’s name to get their attention.
*Protesting*	
Communication of negation, rejection, dislike, or displeasure regarding an object or activity.	Shakes their head to show that they do not want something.
*Indicating Pleasure/Preference*	
Communication of pleasure, acceptance, or interest in an object or activity.	States their likes and dislikes (Example: “I don’t like taking baths, I like showers.”)
*Seeking Information*	
Seeking information from other people about objects, events or interests.	Asks “why” questions (Example: “Why is the playground closed?”)
*Greetings and Goodbyes*	
Prosocial reactions to another person’s entry into or departure from a social situation.	Gives a “high five” or a “fist bump” when a familiar person offers their hand for one.
*Telling About Past Events*	
Reporting information about past events, either by answering questions or reporting spontaneously about an event.	Correctly answers simple questions about what they did that day (Example: “What did you have for lunch today?”)
*Expressing Emotions*	
Expression of positive or negative emotions using actions or words.	Accurately labels their own feelings using words like happy, sad, or mad.
*Communicating Physical States*	
Indicating or reporting about internal physical sensations, such as pain or illness.	Shows, points to, or labels a body part that is sick or in pain (Example: points to ear when asked what hurts).
*Conversation*	
Nonverbal and verbal contributions to interactive, back-and-forth dialogue with other people.	Starts a conversation with another person by making a comment (Examples: “I got new shoes!” or “That’s a cool drawing.”)
*Humor*	
Sharing of positive affect and laughter, especially as it relates to intentional efforts to make “jokes.”	Does things on purpose to make other people laugh (Example: making silly faces).
*Language Clarification & Context*	
Use of strategies to enhance or improve others’ understanding of one’s communication or to better understand others’ communication, either proactively or after a communicative breakdown has occurred.	Provides the needed context or details so that others understand what they are talking about (Example: says, “I got to level 23 in my new video game,” rather than just, “Level 23.”)
*Following Directions*	
Following directions or instructions from others.	Follows directions with three or more steps.
*Imitation*	
Immediately mirroring or copying actions of another person, including gestures, words, or actions with objects.	Mirrors or copies simple play actions (Example: throws a ball into a bucket after the parent does it.)
*Play Skills*	
The variety of ways that children may interact with toys and other objects, including exploration, functional play, and pretend play.	Plays “make believe” with dolls or other toys (Examples: feeds, puts to bed, makes them walk.)
*Social Play with Adults*	
Interactive and reciprocal engagement with adults during play, including initiating, maintaining, and responding.	Asks a familiar adult to play by bringing them a toy or book.
*Understanding Others ’ Thoughts & Emotions*	
Understanding of and response to other people’s thoughts, feelings, and experiences.	Accurately says how another person feels (Example: “Dad is happy.”)
*Social Referencing*	
Orienting to other people (initiation or response) to seek social information, especially in ambiguous situations.	Looks at an object across the room when someone points to it.
*Peer Play*	
Interest in/engagement with similar-aged peers during play and other social interactions.	Asks another child for a turn during play.

**Table 2:** Sociodemographic characteristics of participants in each subgroup

Baseline characteristic	Autistic groups										Typical group	
	Online Retest (*n* = 73)		Online Parent-Parent (*n* = 39)		Online Parent-Teacher (*n* = 37)		Full Online Sample (*n* = 330)		In-Person Validity Sample (*n* = 19)		Full sample (*n* = 401)	
	*n*	%	*n*	*n*	%	%	*n*	%	*n*	%	*n*	%

Child Age in Months (*M*, *SD*, Range)	60.6	12.9	60.1	12.0	62.8	12.3	61.3	12.6	61.2	13.9	46.29	14.7
	37–82		41–82		43–82		33–83		43–82		14–71	
Child Sex at Birth (Male)	51	70	28	72	30	81	165	50	16	84	190	47
Child Race												
Asian	6	8	3	8	1	3	26	8	0	0	10	3
Blacky African American	7	10	2	5	3	8	35	11	2	11	56	14
White	64	88	3	92	34	92	277	84	13	68	323	81
More than one race	7	10	3	8	4	11	37	11	3	16	58	14
Other race	2	2	0	0	2	6	15	6	1	5	15	4
Child Hispanic Ethnicity	9	12	5	13	9	24	66	20	2	11	56	14
Child Language Skills												
Single Words	12	16	6	15	9	24	41	12	3	16	14	4
Short Phrases	23	32	9	23	14	38	102	31	6	32	68	17
Sentences	19	26	12	31	8	22	96	29	6	32	151	38
Complex Speech	19	26	12	31	6	16	91	28	4	21	168	42
Adult Reporter Gender (Male)	7	10	25	64	9	24	23	7	2	11	72	18
Adult is Native English Speaker	66	90	35	90	34	92	302	92	17	89	388	97
Adult Reporter Education												
High School or Less	7	10	6	15	3	8	39	12	2	11	104	26
Some college/Associate’s	22	32	16	41	6	16	113	34	1	5	173	44
Bachelor’s degree	22	30	9	23	17	46	91	28	10	53	81	21
Graduate or professional	21	29	7	18	10	27	87	26	6	32	38	10
Family Income of Child												
<$35,000	12	16	5	13	9	24	59	18	1	5	111	28
$35,000–$74,999	16	22	10	25	7	19	90	27	4	21	172	43
$75,000–$99,999	17	23	11	28	12	32	64	19	4	21	45	11
$100,000 or more	24	33	12	31	8	22	104	32	9	47	63	16
Prefer not to say	4	6	1	3	1	3	13	4	2	11	10	3

**Table 3:** One factor solution for Q-SoCiAL factor analysis (*n* = 330)

Brief Q-SoCiAL item content	Facet	Factor loading

Describes objects using sentences with 4 + words	Commenting	0.94
Asks “how” questions	Seeking Information	0.93
Builds on conversation by answering questions	Conversation	0.92
Asks “why” questions	Seeking Information	0.91
Tells what they want using sentences with 4 + words	Requesting	0.91
Correctly answers simple questions about their day	Telling About Past Events	0.90
Offers multiple details about a topic of interest	Sharing Interest	0.89
Tells multiple details about a past event	Telling About Past Events	0.89
Asks “what” questions	Seeking Information	0.88
States their likes and dislikes	Indicating Pleasure/Preference	0.88
States familiar people’s likes and dislikes	Understanding Others’ Thoughts & Emotions	0.88
Starts a conversation by making a comment	Conversation	0.87
Uses phrases or sentences to indicate pleasure	Indicating Pleasure/Preference	0.87
Describes what is wrong when sick or in pain	Communicating Physical States	0.86
Provides needed context when communicating	Language Clarification/Context	0.86
Explains why they are feeling a certain way	Expressing Emotions	0.85
Responds in the expected way to “small talk”	Conversation	0.83
Actively maintains a conversation for 2 minutes	Conversation	0.83
Accurately says how another person feels	Understanding Others’ Thoughts & Emotions	0.82
Can tell who a pronoun refers to	Understanding Others’ Thoughts & Emotions	0.81
Says their first name when asked	Greetings and Goodbyes	0.81
Uses a familiar person’s name to get their attention	Gaining Attention	0.80
Asks another child for a turn during play	Peer Play	0.79
Accurately labels their own feelings	Expressing Emotions	0.77
Uses words or gestures to ask for turn from an adult	Social Play with Adults	0.76
Says “mama” or “dada” for attention	Gaining Attention	0.75
Able to solve small conflicts with other children	Peer Play	0.75
Shows or points to body part that is sick/in pain	Communicating Physical States	0.72
Says sorry when they accidentally hurt someone	Understanding Others’ Thoughts & Emotions	0.72
Describes the qualities of objects	Commenting	0.72
Takes turns with adults during play	Social Play with Adults	0.71
Matches facial expressions with emotion words	Understanding Others’ Thoughts & Emotions	0.70
Plays with another child by doing the same activity	Peer Play	0.69
Points at something to share interest with an adult	Sharing Interest	0.69
Imitates other children during play	Peer Play	0.68
Tries to comfort someone who is sad	Understanding Others’ Thoughts & Emotions	0.68
Does things on purpose to make other people laugh	Humor	0.67
Follows directions with three or more steps	Following Directions	0.66
Uses the word “no”	Protesting	0.65
Offers pretend food or drink to another person	Play Skills	0.64
Mirrors or copies two or more actions in a row	Imitation	0.61
Plays “make believe” with dolls or other toys	Play Skills	0.60
Mirrors or copies simple play actions	Imitation	0.59
Follows one-step directions	Following Directions	0.55
Names toys or pictures using single words	Commenting	0.39
Plays with toys in the intended way	Play Skills	0.38

**Table 4:** Correlations between Q-SoCiAL and other measures

*N* = 19	Zero-order correlation	Partial correlation (controlling for nonverbal IQ)

Nonverbal IQ	0.59**	–
Verbal IQ	0.64**	0.35
Full Scale IQ	0.65**	0.37
VABS Communication	0.60**	0.28
VABS Socialization	0.59**	0.51*
VABS Daily Living	0.30	0.04
VABS Motor	0.37	0.19
VABS Composite	0.56**	0.33
CARS-2	−0 77***	−0.61**
Behavior Inflexibility	−0.01	0.03

* *p* <. 05

** *p* < .01

*** *p* < .001 (uncorrected)

CARS-2 = Childhood Autism Rating Scales, 2nd Edition (t-score); IQ = Intelligence Quotient (standard score); Q-SoCiAL = Questionnaire for Social Communication in Young Autistic Learners (raw total score); VABS = Vineland Adaptive Behavior Scales, 3rd Edition (scaled/standard score)
