# Supplementary material for: Development and Psychometric Validation of the Questionnaire for Social Communication in Young Autistic Learners (Q-SoCiAL)-Verbal Version
Source: J Autism Dev Disord. Author manuscript; Available in PMC 2026 Aug 31. (PMC13527689; doi:10.1007/s10803-026-07444-8)
Supplement: Supplementary file 1 [file NIHMS2201760-supplement-Appendix_A_Measure_List.docx]

**Appendix A.**

*List of Reviewed Measures*

1. Adaptive Behavior Assessment System Third Edition; Parent/Primary Caregiver Form
2. Adaptive Behavior Assessment System Third Edition; Teacher/Daycare Provider Form
3. Adaptive Behavior Diagnostic Scale (ABDS)
4. Autism Diagnostic Interview-Revised
5. Ages and Stages Questionnaire, 3rd edition
6. Ages and Stages Questionnaire, Social-Emotional, 2^nd^ Edition
7. Autism Spectrum Rating Scales
8. Autism Impact Measure
9. Autism Interview Template
10. Autism Social Skills Profile
11. Autism Treatment Evaluation Checklist
12. Behavioral and Emotional Parent Rating Scale - Second Edition
13. Behavioral and Emotional Teacher Rating Scale- Second Edition
14. Behavior Rating Inventory of Executive Function
15. Child Behavior Checklist for Ages 1.5-5
16. Child Behavior Rating Scale
17. Child Interview of Social Functioning
18. Communication Matrix
19. Conners Early Childhood Teacher/Childcare Provider
20. Communication and Symbolic Behavior Scales-Developmental Profile
21. Conversational Skills Rating Scale
22. Developmental Assessment of Young Children
23. Developmental Profile 3
24. Early Childhood Behavior Scale
25. Eyeberg Child Behavior Inventory
26. Gilliam Asperger's Disorder Scale
27. Gilliam Autism Rating Scale -Third Edition
28. Infant Toddler Development Assessment- Second Edition
29. Infant Toddler Social Emotional Assessment
30. Language Use Inventory
31. MacArthur-Bates Communicative Development Inventories, Words and Gestures
32. Modified Checklist for Autism in Toddlers, Revised with Follow-Up
33. Matson Evaluation of Social Skills with Youngsters-II
34. Matson Evaluation of Social Skills with Youngsters
35. Nisonger Child Behavior Rating Form - Teacher
36. Nisonger Child Behavior Rating Form – Parent
37. NIH Toolbox Item Bank
38. NIH Toolbox Parent Report Negative Parent Relations
39. NIH Toolbox Parent Report Peer Interactions
40. NIH Toolbox Parent Report Peer Rejection
41. NIH Toolbox Parent Report Social Withdrawal
42. Parent Interview of Social Functioning
43. Parents Evaluation of Developmental Status
44. Pediatric Evaluation of Disability Inventory, Version 1.0
45. Pictoral Infant Communication Scales
46. Preschool Language Scales Fifth Edition, home communication questionnaire
47. Preschool and Kindergarten Behavior Scales
48. Preschool and Kindergarten Behavior Scales Second Edition
49. Preschool Behavior Questionnaire
50. Preschool Social Skills Questionnaire
51. Profile of Social Difficulty
52. PROMIS_Parent_Proxy_Bank_v.1.0_-_Family_Relationships_9-5-2017
53. PROMIS_Parent_Proxy_Bank_v2.0_-_Peer_Relationships_7-29-2016
54. PROMIS_Parent_Proxy_SF_v.1.0_-_Family_Relationships_8a_9-5-2017
55. PROMIS_Parent_Proxy_SF_v2.0_-_Peer_Relationship_7a_7-22-2019
56. School Companion-Sensory Profile 2
57. Social Behavior Scales – II
58. Social Communication Questionnaire
59. Social Skills Improvement System - Parent
60. Social Skills Improvement System - Teacher
61. Bayley Scales of Infant and Toddler Development Third Edition, Social-Emotional
62. Bayley Scales of Infant and Toddler Development Third Edition, Adaptive Behavior Questionnaire
63. Stage 1 PDDST II, pervasive developmental disorders test- II
64. Stage 2 PDDST II, developmental clinic screener (DCS)
65. Stage 3 PDDST II, Autism Clinic Severity Screener
66. Teacher Interview of Social Functioning
67. The Rossetti Infant Toddler Language Scale
68. The Rutter Children’s Behaviour Questionnaire
69. Vineland Adaptive Behavior Scales Third Edition
70. Social Responsiveness Scale, 2^nd^ Edition
