# Supplementary material for: Development and Psychometric Validation of the Questionnaire for Social Communication in Young Autistic Learners (Q-SoCiAL)-Verbal Version
Source: J Autism Dev Disord. Author manuscript; Available in PMC 2026 Aug 31. (PMC13527689; doi:10.1007/s10803-026-07444-8)
Supplement: Supplementary file 2 [file NIHMS2201760-supplement-Table_S1_Item_Facet_Coverage.docx]

**Table S1**

*Item Facet Coverage on Each Reviewed Source*

| Facet |  | Present in Source | | | | | |
| --- | --- | --- | --- | --- | --- | --- | --- |
|  |  | Focus Groups | Existing Measures |  | Explicitly Included in Key Theoretic Frameworks? | | |
|  |  |  |  |  | DSM-5 | ESCS | VABS-3 |
| Requesting |  | X | X |  | No | X | Exp Lan |
| Commenting |  | X | X |  | X | X | Exp Lan |
| Sharing Interest |  | X | X |  | X | X | Exp Lan  Int Rel |
| Gaining Attention |  | X | X |  | X | No | Exp Lan |
| Protesting |  | X | X |  | No | X | Exp Lan |
| Indicating Pleasure/Preference |  | X | X |  | No | No | Exp Lan |
| Seeking Information |  | X | X |  | No | No | Exp Lan |
| Greetings and Goodbyes |  | X | X |  | X | No | Exp Lan  Int Rel |
| Telling About Past Events |  | X | X |  | No | No | Exp Lan |
| Expressing Emotions |  | X | X |  | X | No | Exp Lan  Int Rel |
| Communicating Physical States |  | X | X |  | No | No | No |
| Conversation |  | X | X |  | X | No | Int Rel |
| Humor |  | X | No |  | No | No | No |
| Language Clarification & Context |  | X | X |  | X | No | Exp Lan |
| Following Directions |  | X | X |  | No | X | Rec Lan |
| Imitation |  | X | X |  | X | X | Int Rel |
| Play Skills |  | X | X |  | X | No | Play Leis |
| Social Play with Adults |  | X | X |  | X | X | Play Leis |
| Understanding Others’ Thoughts & Emotions |  | X | X |  | X | No | Int Rel |
| Social Referencing |  | X | X |  | X | X | Rec Lan  Coping |
| Peer Play |  | X | X |  | X | No | Int Rel  Play Leis |

DSM-5 = Diagnostic and Statistical Manual of Mental Disorders, 5^th^ Edition Text Revision; ESCS = Early Social Communication Scales; VABS-3= Vineland Adaptive Behavior Scales, 3^rd^ Edition; Exp Lan = Expressive Language subdomain; Rec Lang = Receptive Language subdomain; Int Rel = Interpersonal Relationships subdomain; Play Leis = Play & Leisure subdomain; Coping = Coping Skills subdomain
