# Supplementary material for: Development and Psychometric Validation of the Questionnaire for Social Communication in Young Autistic Learners (Q-SoCiAL)-Verbal Version
Source: J Autism Dev Disord. Author manuscript; Available in PMC 2026 Aug 31. (PMC13527689; doi:10.1007/s10803-026-07444-8)
Supplement: Supplementary file 3 [file NIHMS2201760-supplement-Table_S2_Fit_Statistics.docx]

**Table S2**

*Model Fit for Exploratory Factor Analysis*

| Model Factor Solution | c^2^ | *df* | RMSEA | | TLI | SRMR |
| --- | --- | --- | --- | --- | --- | --- |
|  |  |  | Value | 90% CI |  |  |
| One factor | 3005.30 | 989 | 0.079 | 0.076 - 0.082 | 0.945 | 0.083 |
| Two factor | 2042.68 | 944 | 0.059 | 0.056 - 0.063 | 0.969 | 0.053 |
| Three factor | 1800.64 | 900 | 0.055 | 0.051 - 0.059 | 0.973 | 0.046 |

*Note.* df = degrees of freedom; RMSEA = Root Mean Square Error of Approximation; TLI = Tucker-Lewis Index; SRMR = Standardized Root Mean Square Residual
