# Supplementary material for: Development and Psychometric Validation of the Questionnaire for Social Communication in Young Autistic Learners (Q-SoCiAL)-Verbal Version
Source: J Autism Dev Disord. Author manuscript; Available in PMC 2026 Aug 31. (PMC13527689; doi:10.1007/s10803-026-07444-8)
Supplement: Supplementary file 4 [file NIHMS2201760-supplement-Table_S3_Item_Characteristics.docx]

**Table S3**

*Item Characteristics for Retained Q-SoCiAL Items*

| Brief Q-SoCiAL Item Content |  | Mean | Standard Deviation | Scores at Min (%) | Scores at Max (%) | Item-Total Correlation |
| --- | --- | --- | --- | --- | --- | --- |
| names toys or pictures using single words |  | 2.69 | 0.68 | 3 | 79 | 0.26 |
| uses the word “no” |  | 2.61 | 0.77 | 4 | 74 | 0.50 |
| says “mama” or “dada” for attention |  | 2.60 | 0.88 | 7 | 80 | 0.50 |
| follows one-step directions |  | 2.58 | 0.76 | 5 | 71 | 0.44 |
| plays with toys in the intended way |  | 2.57 | 0.70 | 2 | 67 | 0.29 |
| mirrors or copies simple play actions |  | 2.45 | 0.76 | 4 | 58 | 0.47 |
| uses a familiar person’s name to get their attention |  | 2.33 | 1.06 | 12 | 66 | 0.64 |
| describes the qualities of objects |  | 2.24 | 1.04 | 12 | 57 | 0.61 |
| matches facial expressions with emotion words |  | 2.21 | 1.03 | 13 | 53 | 0.61 |
| offers pretend food or drink to another person |  | 2.17 | 1.04 | 12 | 52 | 0.56 |
| mirrors or copies two or more actions in a row |  | 2.12 | 1.02 | 12 | 46 | 0.53 |
| points at something to share interest with an adult |  | 2.10 | 1.06 | 12 | 50 | 0.62 |
| plays “make believe” with dolls or other toys |  | 2.09 | 1.04 | 13 | 46 | 0.52 |
| says their first name when asked |  | 2.06 | 1.24 | 23 | 57 | 0.69 |
| shows or points to body part that is sick/in pain |  | 2.06 | 1.05 | 14 | 44 | 0.65 |
| plays with another child by doing the same activity |  | 2.00 | 0.93 | 9 | 33 | 0.59 |
| does things on purpose to make other people laugh |  | 1.94 | 1.05 | 16 | 37 | 0.61 |
| imitates other children during play |  | 1.92 | 0.95 | 11 | 31 | 0.58 |
| tells what they want using sentences with 4+ words |  | 1.88 | 1.27 | 26 | 50 | 0.80 |
| asks “what” questions |  | 1.83 | 1.31 | 29 | 49 | 0.78 |
| takes turns with adults during play |  | 1.82 | 1.11 | 20 | 34 | 0.62 |
| uses words or gestures to ask for turn from an adult |  | 1.81 | 1.16 | 22 | 37 | 0.67 |
| accurately labels their own feelings |  | 1.76 | 1.18 | 24 | 36 | 0.70 |
| uses phrases or sentences to indicate pleasure |  | 1.75 | 1.23 | 26 | 40 | 0.79 |
| describes objects using sentences with 4+ words |  | 1.73 | 1.28 | 30 | 43 | 0.84 |
| tries to comfort someone who is sad |  | 1.71 | 1.10 | 20 | 30 | 0.62 |
| states their likes and dislikes |  | 1.69 | 1.27 | 30 | 40 | 0.78 |
| accurately says how another person feels |  | 1.61 | 1.22 | 31 | 32 | 0.76 |
| describes what is wrong when sick or in pain |  | 1.52 | 1.21 | 32 | 28 | 0.80 |
| builds on conversation by answering questions |  | 1.47 | 1.26 | 38 | 29 | 0.84 |
| says sorry when they accidentally hurt someone |  | 1.44 | 1.22 | 35 | 27 | 0.66 |
| can tell who a pronoun refers to |  | 1.44 | 1.24 | 36 | 29 | 0.74 |
| correctly answers simple questions about their day |  | 1.42 | 1.22 | 35 | 27 | 0.81 |
| asks “why” questions |  | 1.39 | 1.35 | 43 | 36 | 0.76 |
| asks another child for a turn during play |  | 1.30 | 1.12 | 35 | 16 | 0.74 |
| offers multiple details about a topic of interest |  | 1.26 | 1.28 | 43 | 36 | 0.77 |
| starts a conversation by making a comment |  | 1.22 | 1.23 | 44 | 23 | 0.77 |
| follows directions with three or more steps |  | 1.21 | 1.09 | 37 | 14 | 0.78 |
| explains why they are feeling a certain way |  | 1.15 | 1.17 | 44 | 18 | 0.76 |
| asks “how” questions |  | 1.13 | 1.26 | 49 | 24 | 0.78 |
| responds in the expected way to “small talk” |  | 1.12 | 1.19 | 47 | 18 | 0.73 |
| states familiar people’s likes and dislikes |  | 1.12 | 1.23 | 49 | 22 | 0.76 |
| tells multiple details about a past event |  | 1.05 | 1.15 | 48 | 15 | 0.76 |
| provides needed context when communicating |  | 0.85 | 1.12 | 59 | 12 | 0.70 |
| able to solve small conflicts with other children |  | 0.79 | 0.95 | 53 | 5 | 0.65 |
| actively maintains a conversation for 2 minutes |  | 0.78 | 1.02 | 58 | 7 | 0.68 |

0 = “Never” or “Only with Help”; 1 = “Rarely”; 2= “Sometimes”; 3 = “Often” or “Outgrown”
